# A Simplified Method for Evaluating Chitin-Binding Activity Applied to YKL-40 (HC-gp39, CHI3L1) and Chitotriosidase

**DOI:** 10.3390/molecules30010019

**Published:** 2024-12-25

**Authors:** Keita Suzuki, Hidetoshi Suzuki, Ami Tanaka, Miwa Tanaka, Kairi Takase, Hiromu Takei, Tomoki Kanaizumi, Kazuaki Okawa, Peter O. Bauer, Fumitaka Oyama

**Affiliations:** 1 Department of Chemistry and Life Science, Kogakuin University, Tokyo 192-0015, Japan; bd22004@ns.kogakuin.ac.jp (K.S.); bm20026@g.kogakuin.jp (H.S.); s121046@g.kogakuin.jp (A.T.); s121047@g.kogakuin.jp (M.T.); s121044@g.kogakuin.jp (K.T.); hiromu0227soccer@gmail.com (H.T.); bm22013@g.kogakuin.jp (T.K.); st13649@ns.kogakuin.ac.jp (K.O.); 2 Bioinova a.s., Videnska 1083, 142 00 Prague, Czech Republic; peter.bauer@bioinova.cz

**Keywords:** catalytic domain (CatD), chitin, chitin-binding activity, chitin-binding affinity assay, chitin-binding domain (CBD), chitotriosidase (CHIT1), chitinase-like proteins (CLPs), W69 residue, YKL-39, YKL-40

## Abstract

YKL-40 is structurally similar to chitotriosidase (CHIT1), an active chitinase, but it lacks chitin-degrading activity while retaining chitin-binding capability. Elevated YKL-40 levels are associated with inflammatory diseases and cancers, making it a valuable biomarker. We previously reported that the W69T substitution in YKL-40 significantly reduces its chitin-binding affinity, identifying W69 as a crucial binding site. In this study, we establish a novel chitin-binding affinity evaluation method using a three-step buffer system to assess the binding strength and specificity of chitin-binding proteins and apply it to characterize YKL-40’s binding mechanism. Our findings confirm that YKL-40, through its key residue W69, exhibits highly specific and robust affinity to chitin. Unlike CHIT1, which has both a catalytic domain (CatD) and a chitin-binding domain (CBD) that allow for diverse binding and degradation activities, YKL-40 lacks a CBD and is specialized for specific chitin recognition without degrading it. Comparative analysis with YKL-39, which does not contain a corresponding W69 residue, highlights the unique role of this residue in YKL-40’s chitin-binding activity that is potentially linked to immune and inflammatory responses. Our evaluation method clarifies YKL-40’s binding properties and provides a versatile approach applicable to other chitin-binding proteins.

## 1. Introduction

Chitin is a polysaccharide composed of *N*-acetyl-D-glucosamine (GlcNAc) units linked by β-1,4 bonds, serving as a crucial structural component in the exoskeletons of crustaceans and insects, the cell walls of fungi, and the microfilarial sheath of parasitic nematodes [1,2,3]. Although mammals do not synthesize chitin, they produce chitinases, such as chitotriosidase (CHIT1) and acidic chitinase (CHIA), with degradation abilities [4,5,6,7].

In addition to chitinases, mammals, including humans, express chitinase-like proteins (CLPs), which share structural similarities with chitinases but lack enzymatic activity [1,3,8,9,10,11,12,13,14,15,16,17,18,19,20,21]. Among these CLPs, YKL-40 (CHI3L1) and YKL-39 (CHI3L2) are well studied. YKL-40 was initially identified in the conditioned medium of human osteoblasts and named after the first three amino acids in its N-terminus (tyrosine, lysine, and leucine: YKL), with a molecular weight of approximately 40 kDa [11,22,23]. Structurally, YKL-40 shares similarities with the catalytic domain (CatD) of human CHIT1, yet lacks chitinase activity. Notably, elevated levels of YKL-40 are observed in a variety of conditions, including asthma, chronic obstructive pulmonary disease (COPD), inflammatory bowel disease (IBD), alcoholic cirrhosis, Alzheimer’s disease, and several types of cancer [24,25,26,27,28,29,30,31,32,33,34,35,36], indicating its potential as a biomarker [3]. Therefore, understanding its pathophysiological role is of considerable importance.

Our previous research identified W69 in YKL-40 as a critical residue for substrate recognition, highlighting its significance in the functional role of YKL-40 [37]. Although YKL-40 is structurally similar to CHIT1, it lacks chitinase activity, which is influenced by catalytic motif substitutions and sequence modifications that affect chitin-binding affinity. Our earlier findings suggested that YKL-40 may exhibit stronger chitin-binding activity than the CatD of CHIT1 [37].

In contrast to YKL-40, CHIT1 is a full-length chitinase that includes a CatD and a chitin-binding domain (CBD) [5,6,38,39]. While the CBD anchors and stabilizes the chitin substrate, the CatD facilitates enzymatic cleavage. However, the distinct contributions of these domains to CHIT1’s degradative function still need to be explored.

Traditional methods for assessing chitin-binding activity, such as single-buffer elution techniques, provide only binary outcomes (bound/unbound) and lack the resolution to differentiate binding strengths or modes [37,39,40,41,42]. Advanced techniques, such as isothermal titration calorimetry (ITC) and surface plasmon resonance (SPR), provide detailed thermodynamic and kinetic data [21,43,44], but these are expensive and time-consuming methods that are better suited for low-molecular-weight substrates like chitin oligosaccharides. Moreover, these methods face challenges in analyzing interactions with high-molecular-weight chitin.

This study introduces a novel chitin-binding assay using a three-step buffer system. This approach enables the stepwise evaluation of binding strength and specificity, overcoming the limitations of conventional methods. By applying this assay, we aimed to characterize the chitin-binding properties of YKL-40, CHIT1, and YKL-39. Our findings provide insights into the molecular mechanisms underlying protein–chitin interactions and establish a practical tool for advancing research on CLPs, chitinases, and related biomolecular systems.

## 2. Results

### 2.1. Improved Quantitative Method for Assessing Chitin-Binding Activity

**Figure 1:**
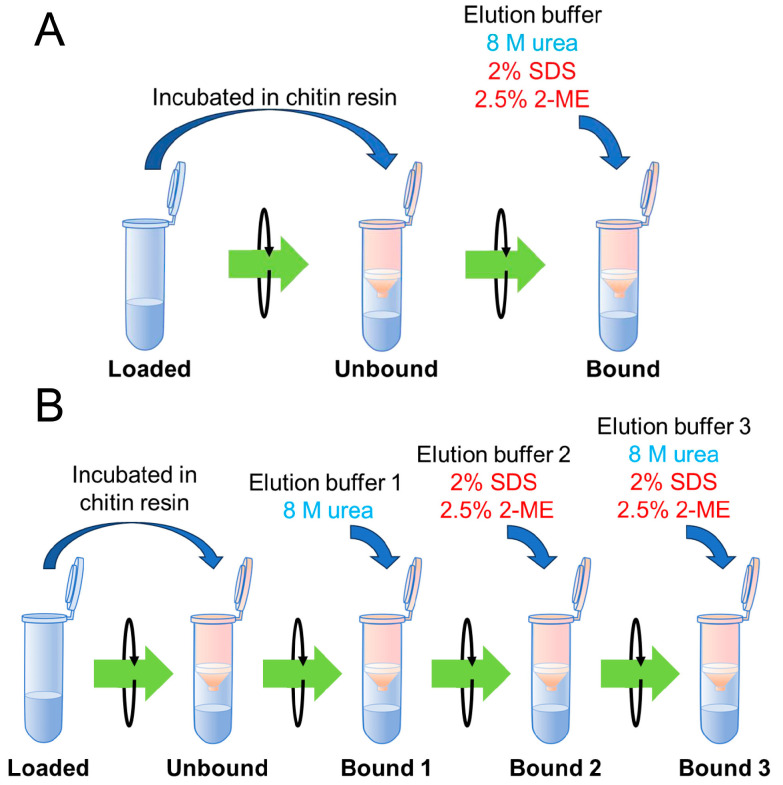
A comparison of conventional and improved chitin-binding methods. (**A**) The conventional chitin-binding method. Protein samples are incubated with chitin resin to allow binding. After incubation, the unbound fraction is collected by centrifugation, and the resin-bound proteins are eluted using a single buffer (8 M urea/2% SDS/2.5% 2-ME). This one-step elution method provides basic information on the presence or absence of binding. (**B**) The improved chitin-binding method. In this enhanced protocol, proteins are incubated with chitin resin, and the unbound fraction is collected. To evaluate binding strength more precisely, bound proteins are sequentially eluted using three different buffers: 8 M urea (Buffer 1), 2% SDS/2.5% 2-ME (Buffer 2), and 8 M urea/2% SDS/2.5% 2-ME (Buffer 3). This multi-step elution allows for a stepwise assessment of binding strength under various conditions.

In a recent study, proteins bound to chitin resin were treated with a single type of elution buffer [8 M urea, 2% SDS, and 2.5% 2-mercaptoehanol (ME)] [37]. In this conventional method, proteins incubated with chitin resin were eluted with a single buffer, and binding ability was evaluated by comparing the unbound and bound fractions (Figure 1A). While this approach provided clear results regarding the presence of chitin-binding proteins, it was insufficient for assessing the strength of chitin-binding.

To explore whether proteins could be differentiated based on their binding modes to chitin, we divided the conventional elution buffer into three types according to their components. This allowed us to classify the chitin-bound fractions into three categories (Figure 1B).

Specifically, 8 M urea was used as Buffer 1; 2% SDS with 2.5% 2-ME was used as Buffer 2; and a mixture of urea, SDS, and 2-ME was used as Buffer 3. Urea is believed to disrupt hydrogen and hydrophobic bonds, while SDS and 2-ME break hydrophobic and disulfide bonds. Buffer 3 is expected to disrupt all of these interactions.

### 2.2. Evaluation of the Improved Chitin-Binding Activity Assay

**Figure 2:**
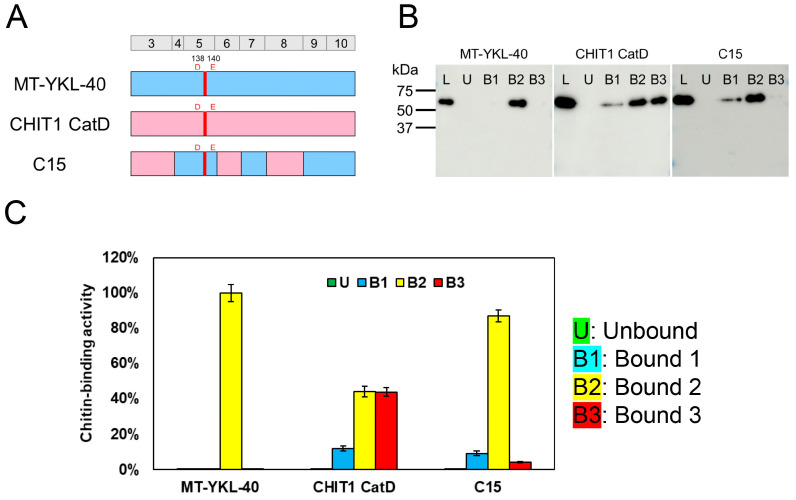
Evaluation of chitin-binding activity using MT-YKL-40, CHIT1 CatD, and chimeric protein C15. (**A**) Schematic of proteins used in the assay: MT-YKL-40, CHIT1 CatD, and the chimeric protein C15 (combining MT-YKL-40 and CHIT1 CatD regions). MT-YKL-40 contains A138D and L140E substitutions in WT-YKL-40. Color-coding: pink, CHIT1 CatD; blue, YKL-40 sequence; red, active motif introduced by the A138D and L140E mutations in WT-YKL-40. (**B**) Western blot analysis: Recombinant proteins were analyzed for binding to chitin resin at pH 7.0. Fractions: L (loaded), U (unbound), B1 (Bound 1), B2 (Bound 2), and B3 (Bound 3). (**C**) Quantification of chitin-binding activity. Bound fractions were quantified, with L (loaded fraction) set to 100%. Graph colors: U (green), B1 (light blue), B2 (yellow), and B3 (red). Error bars represent the mean ± SD from a single experiment conducted in triplicate.

Using the presented improved methods, the unbound fraction was first collected for each sample, followed by up to three bound fractions, as shown in Figure 1B and de-scribed in Materials and Methods. This new experimental approach was verified using mutated YKL-40 (MT-YKL-40), which was constructed by introducing A138D and L140E mutations in the wild-type protein (WT-YKL-40), CHIT1 CatD, and the chimeric protein C15 (Figure 2A and Supplementary Figures S1 and S2), which consists of CHIT1 CatD and MT-YKL-40, previously confirmed to have chitinase activity [37]. The A138D and L140E mutations in MT-YKL-40 were specifically designed to mimic residues found in CHIT1, conferring chitinase-like properties. The results are presented in Figure 2B.

For MT-YKL-40, elution was observed exclusively in the B2 fraction (Figure 2B,C). For CHIT1 CatD, elution occurred in approximately 10% in the B1 fraction and 40% in both the B2 and B3 fractions. For the chimeric protein C15, elution was primarily in the B2 fraction, with additional bands detected in the B1 and B3 fractions. These findings indicate that MT-YKL-40 has a strong substrate recognition ability, while CHIT1 CatD exhibits diverse binding modes and strengths, suggesting a broader range of chitin-binding interactions. The chimeric protein C15 displayed intermediate binding properties, combining characteristics of both MT-YKL-40 and CHIT1 CatD.

These results highlight the utility of the improved elution method in differentiating chitin-binding activities based on both binding modes and strengths, providing a more nuanced understanding of protein–chitin interactions.

### 2.3. Evaluation of Chitin-Binding in Full-Length CHIT1, CatD, and CBD Using the New Method

**Figure 3:**
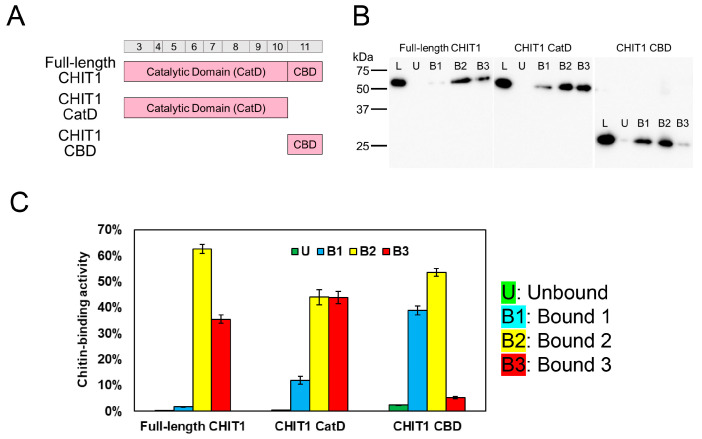
Chitin-binding analysis of CHIT1 domains. (**A**) Schematic representation of full-length CHIT1 and its domains. CHIT1 includes a catalytic domain (CatD) and a chitin-binding domain (CBD). (**B**) Western blot analysis. Recombinant proteins were analyzed for binding to chitin resin at pH 7.0. Fractions: L (loaded), U (unbound), B1 (Bound 1), B2 (Bound 2), and B3 (Bound 3). (**C**) Quantification of chitin-binding activity. Bound fractions were quantified, with L set to 100%. Graph colors: U (green), B1 (light blue), B2 (yellow), and B3 (red). Error bars represent the mean ± SD from a single experiment conducted in triplicate.

Using this new method, we further evaluated the chitin-binding abilities of each domain within CHIT1. The full-length CHIT1 protein comprises a catalytic domain (CatD) and a chitin-binding domain (CBD) (Figure 3A and Supplementary Figures S1 and S2). We examined whether these domains exhibit differences in chitin-binding strength.

The full-length CHIT1 protein showed limited elution in the B1 fraction, with higher elution in the B2 fraction, indicating strong binding through hydrophobic and/or disulfide bonds (Figure 3B,C). CatD alone exhibited similar binding properties, as described in Figure 2B,C. In contrast, the CBD showed increased elution in the B1 fraction and reduced elution in the B3 fraction, suggesting that its binding affinity is weaker through hydrogen and/or hydrophobic than that of CatD.

These results suggest that CHIT1 CatD exhibits diverse binding patterns that may facilitate substrate degradation. CBD, on the other hand, may support CatD by enhancing substrate recognition and binding. Together, in the full-length CHIT1, these domains combine their respective properties, resulting in powerful substrate recognition and effective control over binding and dissociation with substrates.

These findings also indicate that our newly developed chitin-binding evaluation method is effective for assessing the binding properties of the CHIT1 domains.

### 2.4. Analysis of the W69 Mutation in YKL-40 Using the New Evaluation Method

Next, we used our evaluation method to analyze the effect of the W69 mutation in YKL-40. Our previous research has shown that introducing the W69T substitution into MT-YKL-40 significantly reduces chitin-binding ability, highlighting the importance of W69 in chitin substrate recognition [37]. In this study, we applied the chitin-binding analysis method established here to further investigate the impact of the W69T substitution on YKL-40.

**Figure 4:**
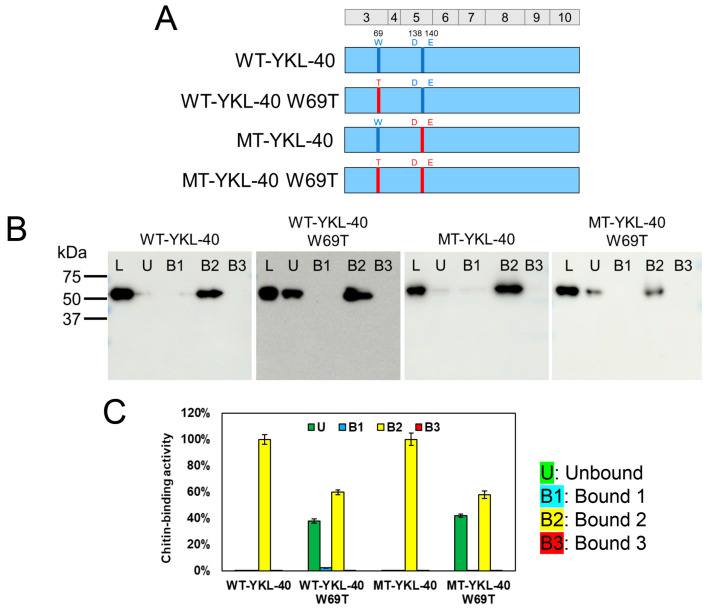
The effect of W69 mutation on YKL-40 binding. (**A**) Schematic representation of YKL-40 with/without W69T mutation. WT-YKL-40 and MT-YKL-40 were constructed to assess W69T’s effect. Color-coding: blue, YKL-40 sequence (inactive motif); red, active motif introduced by the A138D and L140E mutations in WT-YKL-40. (**B**) Western blot analysis, showing chitin resin binding at pH 7.0. Fractions: L, U, B1, B2, and B3. (**C**) Quantification of chitin-binding activity. Bound fractions were quantified, with L set to 100%. Graph colors: U (green), B1 (light blue), B2 (yellow), and B3 (red). Error bars represent the mean ± SD from a single experiment conducted in triplicate.

For this purpose, we created WT-YKL-40, which lacks the catalytic motif with an introduced W69 substitution, as well as MT-YKL-40 with an introduced catalytic motif and the W69T substitution (Figure 4A and Supplementary Figures S1 and S2). For both WT- and MT-YKL-40, before the W69T substitution, bands were detected only in the B2 fraction, indicating strong binding through hydrophobic and/or disulfide bonds (Figure 4B,C). However, with the introduction of the W69T substitution, clear bands also appeared in the unbound fractions, indicating a reduction in binding ability.

### 2.5. Evaluation of the K74W Substitution in YKL-39 Using the New Method

Since previous research suggested that YKL-40 possesses a more extended substrate-binding site than YKL-39 [21], we investigated whether introducing W at the 74th position in YKL-39, corresponding to W69 in YKL-40, could alter its chitin-binding properties.

**Figure 5:**
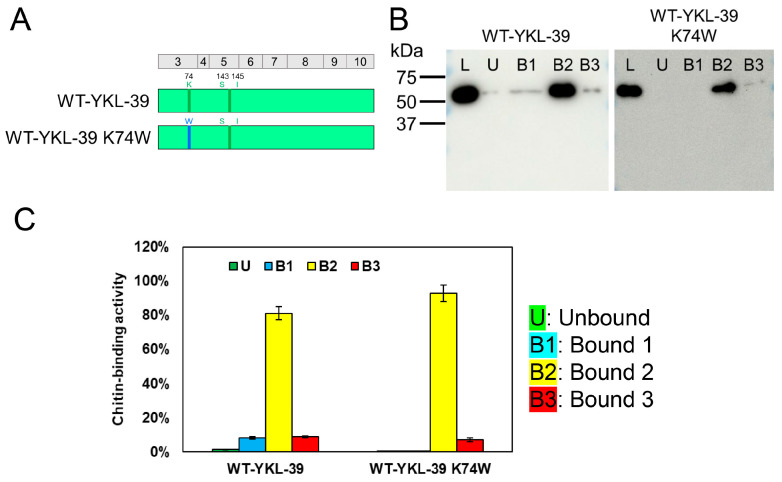
The effect of K74W substitution on YKL-39 binding. (**A**) Schematic representation of WT-YKL-39 and WT-YKL-39 K74W. WT-YKL-39 and K74W-substituted YKL-39 were analyzed. K74 (YKL-39) corresponds to W69 (YKL-40). Color-coding: light green, YKL-39 sequence (including residues S143 and I145 of the inactive motif); blue, K74W mutation introduced into WT-YKL-39, corresponding to W69 in YKL-40. (**B**) Western blot analysis, showing chitin resin binding at pH 7.0. Fractions: L, U, B1, B2, and B3. (**C**) Quantification of chitin-binding activity. Bound fractions were quantified, with L set to 100%. Graph colors: U (green), B1 (light blue), B2 (yellow), and B3 (red). Error bars represent the mean ± SD from a single experiment conducted in triplicate.

We replaced this amino acid in wild-type proteins (WT-YKL-39) and evaluated the effect on chitin binding (Figure 5A and Supplementary Figures S1 and S2). WT-YKL-39 primarily eluted in the B2 fraction, with slight elution observed in the B1 and B3 fractions (Figure 5B,C). However, after the K74W substitution, no elution was observed in the B1 fraction, and the chitin-bound portion in the B2 fraction increased. This suggests that introducing W at position 74 enhances the strength and specificity of chitin binding, likely by providing additional hydrophobic or π-π stacking interactions. These changes mirror the binding properties observed in YKL-40, indicating that the K74W substitution confers functional characteristics similar to W69 in YKL-40.

## 3. Discussion

In this study, we developed a novel method to evaluate chitin-binding activity, enabling the stepwise assessment of binding strength and specificity across different protein domains. Unlike conventional single-buffer methods [37,39,40,41,42], which provide binary outcomes (bound/unbound) and fail to resolve interaction types, our three-step buffer system enables detailed characterization by sequentially disrupting hydrogen, hydrophobic, and disulfide bonds. This cost-effective approach addresses the key limitations of previous methods and offers a nuanced understanding of protein–chitin interactions.

Our results confirm that YKL-40, despite lacking the CBD present in CHIT1, exhibits highly specific and robust chitin-binding activity, with W69 identified as a key residue mediating substrate recognition. Unlike CHIT1, which has evolved as a chitin-degrading enzyme with diverse binding modes across its CatD and CBD, YKL-40 recognizes the substrate without degrading it. This suggests that YKL-40’s function is likely centered on immune or inflammatory modulation via chitin-like molecules rather than enzymatic breakdown.

Our findings also demonstrate that CHIT1’s CatD and CBD act jointly to process diverse chitin substrates effectively. The CBD likely stabilizes chitin, facilitating its proper positioning for enzymatic cleavage by CatD. This complementary interaction underscores the efficiency of CHIT1’s degradative function and aligns with previous studies emphasizing the concerted roles of these domains.

The comparative analysis with YKL-39 highlights the critical role of W69 in YKL-40’s chitin-binding specificity [37]. YKL-39, which lacks a residue equivalent to W69, exhibited reduced chitin-binding strength and specificity. Interestingly, the K74W substitution in YKL-39 enhanced its binding properties, aligning them more closely with those of YKL-40. These findings delineate how structural adaptations in YKL-40 have contributed to its specialized functional role in recognizing chitin-like molecules.

To further explore the molecular mechanisms underlying protein–chitin interactions, we designed each buffer in our three-step method to disrupt specific types of interactions. Buffer 1 (8 M urea) disrupts hydrogen bonds, affecting polar interactions between proteins and chitin, which are likely mediated by hydrophilic residues. Buffer 2 (2% SDS + 2-mercaptoethanol) disrupts hydrophobic interactions, and 2-mercaptoethanol reduces disulfide bonds, destabilizing aromatic and hydrophobic residues such as W69 in YKL-40. Buffer 3 (8 M urea + SDS + 2-mercaptoethanol) eliminates all these interactions, comprehensively assessing binding strength and specificity.

The distinct elution patterns observed in our assay underscore the importance of hydrophobic and π-π stacking interactions, particularly those mediated by W69 in YKL-40. Docking simulation results with GlcNAc units further validate this specificity. In both WT- and MT-YKL-40, the W69T mutants exhibited slightly higher binding free energies for the lowest-energy binding mode than their respective wild-type proteins (Supplementary Figure S3), indicating reduced binding strength upon W69T substitution. Moreover, the large energy gap between the lowest energy binding mode and the second lowest mode in both WT- and MT-YKL-40 suggests the presence of highly specific binding interactions. In contrast, this energy gap was significantly reduced in the W69T mutants, reflecting increased diversity in binding modes and a corresponding decrease in binding specificity. These findings underscore the critical role of W69 in securely anchoring the ligand within the YKL-40 binding pocket, ensuring high specificity and robust binding interactions. These computational results align with the elution profiles observed in our assay, further substantiating the role of W69 in mediating specific interactions with chitin.

While this study primarily focused on establishing and validating an in vitro chi-tin-binding assay, future research needs to explore the physiological and pathological roles of YKL-40 in vivo. For example, mouse models overexpressing or lacking YKL-40 could be used to assess its effects on cytokine production, immune cell recruitment, or the progression of inflammatory conditions. Studies by Lee et al. demonstrated that breast regression protein 39 (BRP-39, Chi3l1), YKL-40’s murine counterpart, plays critical roles in Th2 inflammation, alternative macrophage activation, and antigen sensitization, highlighting its influence on immune responses [18]. BRP-39 (Chi3l1)-deficient T cells exhibit enhanced Th1 and CTL responses, reducing melanoma lung metastasis by promoting antitumor immunity [45].

Our previous studies quantified BRP-39 (Chi3l1) and YKL-40 expression in mouse and human tissues, indicating their constitutive expression in specific tissues, such as the liver and lung [46,47]. These results suggest tissue-specific roles in maintaining immune homeostasis. These findings establish a foundation for using YKL-40-deficient or transgenic models to study its physiological and pathological contributions to asthma, inflammatory bowel disease, and cancer. These in vivo models would better understand how YKL-40’s chitin-binding activity translates to its functional roles in immune modulation and tissue remodeling.

While the newly developed method provides a simplified and cost-effective approach to assess chitin-binding activity, we acknowledge that advanced techniques such as isothermal titration calorimetry (ITC) and surface plasmon resonance (SPR) offer valuable insights into the thermodynamics and kinetics of protein–ligand interactions [21,43,44]. However, these techniques are better suited for analyzing low-molecular-weight soluble ligands like chitin oligosaccharides. The heterogeneity of high-molecular-weight chitin presents significant challenges for ITC and SPR analysis, making our method particularly valuable for studying interactions with high-molecular-weight substrates.

Additionally, this method demonstrated its adaptability for studying various chitin-binding proteins beyond YKL-40 and CHIT1. Future applications could include other chitinase-like protein (CLP) family members, fungal lectins, and bacterial chitin-binding proteins, which play key roles in diverse biological processes. Furthermore, while this study focused on chitin, the assay could be adapted to study interactions with other polysaccharides, such as cellulose or alginate, by modifying the buffer system.

By integrating this assay with complementary techniques, such as ITC or SPR, future studies could provide a more comprehensive understanding of chitin-binding mechanisms, combining high-throughput analysis with detailed thermodynamic insights [21,43,44]. This study underscores YKL-40’s evolutionary transition from a chitin-degrading enzyme to a recognition protein with specialized roles in immune modulation and inflammatory responses. These findings highlight the broader implications of chitin-binding mechanisms in biological systems and provide a foundation for further research into chitin-binding proteins’ structural and functional adaptations.

## 4. Materials and Methods

### 4.1. Construction of CHIT1, YKL-40, and YKL-39 Vectors for E. coli Expression

Human full-length CHIT1, CHIT1 CatD, and mature WT-YKL-40 cDNAs, along with their chimeric or mutant variants, were prepared as described previously [37,48]. The CHIT1 CBD cDNA was amplified by PCR from full-length CHIT1 using KOD Plus DNA polymerase (Toyobo, Kyoto, Japan). WT-YKL-39 cDNA was synthesized by Eurofins Genomics (Tokyo, Japan). The amplification of cDNAs was performed by PCR using KOD Plus DNA polymerase with oligonucleotide primers designed to incorporate BamHI and XhoI restriction enzyme sites (Supplementary Table S1) [40,49]. The resulting PCR products were cloned into the corresponding sites of the pET22b/pre-Protein A-monkey AMCase-V5-His vector [50].

### 4.2. Preparation of Recombinant YKL-40, CHIT1, and YKL-39 Proteins Expressed in E. coli

The preparation of protein A-CHIT1-V5-His and protein A-YKL-40-V5-His from the *E. coli* was performed and purified by Ni Superflow Resin (Takara Bio, Shiga, Japan), as described previously [37].

### 4.3. SDS-Polyacrylamide Gel Electrophoresis and Western Blot

The protein fractions were analyzed using standard SDS-polyacrylamide gel electrophoresis (PAGE), followed by Western blot, using an anti-V5-HRP monoclonal antibody (Thermo Fisher Scientific, Waltham, MA, USA) [37]. Immunoblots were analyzed and quantified using the Luminescent Image Analyzer (ImageQuant 800, Cytiva, Marlborough, MA, USA) according to the manufacturer’s instructions.

### 4.4. Chitin-Binding Assay

Chitin resin (New England Biolabs, Ipswich, MA, USA) was used for the binding assay and packed into a spin column (Micro Bio-Spin™ Chromatography Columns #7326204; Bio-Rad Laboratories, Hercules, CA, USA). Before binding, the resin was equilibrated with McIlvaine buffer (pH 7.0) containing 0.5 M NaCl. Protein samples were incubated with the resin by adding them to the column, allowing a 1-min incubation at room temperature and centrifugation to collect the flow-through. This process was repeated five times, with the final collected flow-through designated the unbound fraction. Sequential elution steps were conducted using different buffers to evaluate the binding strength. For Bound 1 fraction (B1), 8 M urea in McIlvaine buffer (pH 7.0) containing 0.5 M NaCl was added to the column, followed by a 10-min incubation at room temperature, and the bound proteins were eluted. For Bound 2 fraction (B2), McIlvaine buffer (pH 7.0) containing 0.5 M NaCl, 2% SDS, and 2.5% 2-ME was added and incubated at 37 °C for 30 min before elution. Finally, for Bound 3 fraction (B3), McIlvaine buffer (pH 7.0) containing 0.5 M NaCl, 8 M urea, 2% SDS, and 2.5% 2-ME was added, followed by a 10-min incubation at room temperature, and the final bound proteins were eluted. Each fraction was analyzed by SDS-PAGE and Western blotting, with binding strength quantified based on Western blot band intensities.

### 4.5. Data Analysis

Western blot images were analyzed and quantified using ImageQuant™ TL (ver.8.2, Cytiva). Binding strength was calculated by normalizing band intensities relative to the total input protein [37].

### 4.6. Docking Simulations with GlcNAc Units

The three-dimensional structure of YKL-40 was derived from its crystal structure (PDB accession code 1NWT) [51]. Amino acid substitutions, including W69T, were introduced using ChimeraX [52]. Docking simulations with GlcNAc units were performed using AutoDock Vina (ver.1.2.5, The Scripps Research Institute, La Jolla, CA, USA) [53,54], with binding free energy values and binding modes analyzed to evaluate specificity and strength.

## 5. Conclusions

This study introduces a novel chitin-binding evaluation method that enables the stepwise analysis of binding strength and specificity, overcoming the limitations of conventional binary assays. We used this method to reveal that YKL-40 specializes in chitin recognition, relying on the critical residue W69 for its binding activity. This structural adaptation highlights YKL-40’s evolutionary transition from a chitin-degrading enzyme to a recognition protein, contributing to immune modulation and inflammation regulation.

## Data Availability

Data supporting the reported results will be available from the corresponding author (Fumitaka Oyama).

## Supplementary Materials

The following supporting information can be downloaded at https://www.mdpi.com/article/10.3390/molecules30010019/s1. Figure S1: Deduced amino acid sequences of the recombinant proteins expressed in *E. coli*. Supplementary Figure S2: Western blot analysis of the recombinant proteins using anti-V5 antibody. Supplementary Figure S3: Binding free energy distribution for WT-YKL-40, MT-YKL-40, and their respective W69T mutants. Table S1: Combination of primer and template to prepare the recombinant proteins using PCR.
