# Supplementary material for: A Simplified Method for Evaluating Chitin-Binding Activity Applied to YKL-40 (HC-gp39, CHI3L1) and Chitotriosidase
Source: Molecules. 2024 Dec 25;30(1):19. doi: 10.3390/molecules30010019 (PMC11721955; doi:10.3390/molecules30010019)
Supplement: Supplementary file 1 [file molecules-30-00019-s001.zip › molecules-3358153-supplementary.pdf]

A Simplified Method for Evaluating Chitin-Binding Activity: Application to YKL-40 (HC-gp39, CHI3L1) and Chitotriosidase

Keita Suzuki[1], Hidetoshi Suzuki[1], Ami Tanaka[1], Miwa Tanaka[1], Kairi Takase[1], Hiromu Takei[1], Tomoki Kanaizumi[1], Kazuaki Okawa[1], Peter O. Bauer[2], and Fumitaka Oyama[1]*

Supplementary Figures S1-S3 and Supplementary Table S1.

MT-YKL-40

YKLVCYYTSWSQYREGDGSCFPDALDRFLCTHIIYSFANISNDHIDTWEWNDVTLYGML
NTLKNRNPNLKTLLSVGGWNFGSQRFSKIASNTQSRRTFIKSVPPFLRTHGFDGLDLDW
EYPGRRDKQHFTTLIKEMKAEFIKEAQPGKKQLLLSAALSAGKVTIDSSYDIAKISQHL
DFISIMTYDFHGAWRGTTGHHSPLFRGQEDASPDRFSNTDYAVGYMLRLGAPASKLVMG
IPTFGRSFTLASSETGVGAPISGPGIPGRFTKEAGTLAYYEICDFLRGATVHRILGQQV
PYATKGNQWVGYDDQESVKSKVQYLKDRQLAGAMVWALDLDDFQGSFCGQDLRFPLTNA
IKDALAAT

CHIT1 CatD

AKLVCYFTNWAQYRQGEARFLPKDLDPSLCTHLIYAFAGMTNHQLSTTEWNDETLYQEF
NGLKKMNPKLKTLLAIGGWNFSTQKFTDMVATANNRQTFVNSAIRFLRKYSFDGLDLDW
EYPGSQGSPAVDKERFTTLVQDLANAFQQEAQTSGKERLLLSAAVPAGQTYVDAGYEVD
KIAQNLDFVNLMAYDFHGSWEKVTGHNSPLYKRQEESGAAASLNVDAAVQQWLQKGTPA
SKLILGMPTYGRSFTLASSSDTRVGAPATGSGTPGPFTKEGGMLAYYEVCSWKGATKQR
IQDQKVPYIFRDNQWVGFDDVESFKTKVSYLKQKGLGGAMVWALDLDDFAGFSCNQGRY
PLIQTLRQELSLP

C15

AKLVCYFTNWAQYRQGEARFLPKDLDPSLCTHLIYAFAGMTNHQLSTTEWNDETLYQEF
NGLKKMNPNLKTLLSVGGWNFGSQRFSKIASNTQSRRTFIKSVPPFLRTHGFDGLDLDW
EYPGRRDKQHFTTLIKDLANAFQQEAQTSGKERLLLSAAVPAGQTYVDAGYEVDKIAQH
LDFISIMTYDFHGAWRGTTGHHSPLFRGQEDASPDRFSNTDAAVQQWLQKGTPASKLIL
GMPTYGRSFTLASSSDTRVGAPATGSGTPGPFTKEGGMLAYYEICDFLRGATVHRILGQ
QVPYATKGNQWVGYDDQESVKSKVQYLKDRQLAGAMVWALDLDDFQGSFCGQDLRFPLT
NAIKDALAAT

Full-length CHIT1

AKLVCYFTNWAQYRQGEARFLPKDLDPSLCTHLIYAFAGMTNHQLSTTEWNDETLYQEF
NGLKKMNPKLKTLLAIGGWNFGTQKFTDMVATANNRQTFVNSAIRFLRKYSFDGLDLDW
EYPGSQGSPAVDKERFTTLVQDLANAFQQEAQTSGKERLLLSAAVPAGQTYVDAGYEVD
KIAQNLDFVNLMAYDFHGSWEKVTGHNSPLYKRQEESGAAASLNVDAAVQQWLQKGTPA
SKLILGMPTYGRSFTLASSSDTRVGAPATGSGTPGPFTKEGGMLAYYEVCSWKGATKQR
IQDQKVPYIFRDNQWVGFDDVESFKTKVSYLKQKGLGGAMVWALDLDDFAGFSCNQGRY
PLIQTLRQELSLPYLPSGTPELEVPKPGQPSEPEHGPSPGQDTFCQGKADGLYPNPRER
SSFYSCAAGRLFQQSCPTGLVFSNSCKCCTWN

CHIT1 CBD

PELEVPKPGQPSEPEHGPSPGQDTFCQGKADGLYPNPRERSSFYSCAAGRLFQQSCPTG
LVFSNSCKCCTWN

WT-YKL-40

YKLVCYYTSWSQYREGDGSCFPDALDRFLCTHIIYSFANISNDHIDTWEWNDVTLYGML
NTLKNRNPNLKTLLSVGGWNFGSQRFSKIASNTQSRRTFIKSVPPFLRTHGFDGLDLAW
LYPGRRDKQHFTTLIKEMKAEFIKEAQPGKKQLLLSAALSAGKVTIDSSYDIAKISQHL
DFISIMTYDFHGAWRGTTGHHSPLFRGQEDASPDRFSNTDYAVGYMLRLGAPASKLVMG
IPTFGRSFTLASSETGVGAPISGPGIPGRFTKEAGTLAYYEICDFLRGATVHRILGQQV
PYATKGNQWVGYDDQESVKSKVQYLKDRQLAGAMVWALDLDDFQGSFCGQDLRFPLTNA
IKDALAAT

WT-YKL-40 W69T

YKLVCYYTSWSQYREGDGSCFPDALDRFLCTHIIYSFANISNDHIDTTEWNDVTLYGML
NTLKNRNPNLKTLLSVGGWNFGSQRFSKIASNTQSRRTFIKSVPPFLRTHGFDGLDLAW
LYPGRRDKQHFTTLIKEMKAEFIKEAQPGKKQLLLSAALSAGKVTIDSSYDIAKISQHL
DFISIMTYDFHGAWRGTTGHHSPLFRGQEDASPDRFSNTDYAVGYMLRLGAPASKLVMG
IPTFGRSFTLASSETGVGAPISGPGIPGRFTKEAGTLAYYEICDFLRGATVHRILGQQV

PYATKGNQWVGYDDQESVKSKVQYLKDRQLAGAMVWALDLDDFQGSFCGQDLRFPLTNA
IKDALAAT

MT-YKL-40 W69T
YKLVCYYTSWSQYREGDGSCFPDALDRFLCTHIIYSFANISNDHIDTTEWNDVTLYGML
NTLKNRNPNLKTLLSVGGWNFGSQRFSKIASNTQSRRTFIKSVPPFLRTHGFDGLDLDW
EYPGRRDKQHFTTLIKEMKAEFIKEAQPGKKQLLLSAALSAGKVTIDSSYDIAKISQHL
DFISIMTYDFHGAWRGTTGHHSPLFRGQEDASPDRFSNTDYAVGYMLRLGAPASKLVMG
IPTFGRSFTLASSETGVGAPISGPGIPGRFTKEAGTLAYYEICDFLRGATVHRILGQQV
PYATKGNQWVGYDDQESVKSKVQYLKDRQLAGAMVWALDLDDFQGSFCGQDLRFPLTNA
IKDALAAT

WT-YKL-39
YKLVCYFTNWSQDRQEPGKFTPENIDPFLCSHLIYSFASIENNKVIIKDKSEVMLYQTI
NSLKTKNPKLKILLSIGGYLFGSKGFHPMVDSSTSRLEFINSIILFLRNHNFDGLDVSW
IYPDQKENTHFTVLIHELAEAFQKDFTKSTKERLLLTAGVSAGRQMIDNSYQVEKLAKD
LDFINLLSFDFHGSWEKPLITGHNSPLSKGWQDRGPSSYYNVEYAVGYWIHKGMPSEKV
VMGIPTYGHSFTLASAETTVGAPASGPGAAGPITESSGFLAYYEICQFLKGAKITRLQD
QQVPYAVKGNQWVGYDDVKSMETKVQFLKNLNLGGAMIWSIDMDDFTGKSCNQGPYPLV
QAVKRSLGSL

WT-YKL-39 K74W
YKLVCYFTNWSQDRQEPGKFTPENIDPFLCSHLIYSFASIENNKVIIWDKSEVMLYQTI
NSLKTKNPKLKILLSIGGYLFGSKGFHPMVDSSTSRLEFINSIILFLRNHNFDGLDVSW
IYPDQKENTHFTVLIHELAEAFQKDFTKSTKERLLLTAGVSAGRQMIDNSYQVEKLAKD
LDFINLLSFDFHGSWEKPLITGHNSPLSKGWQDRGPSSYYNVEYAVGYWIHKGMPSEKV
VMGIPTYGHSFTLASAETTVGAPASGPGAAGPITESSGFLAYYEICQFLKGAKITRLQD
QQVPYAVKGNQWVGYDDVKSMETKVQFLKNLNLGGAMIWSIDMDDFTGKSCNQGPYPLV
QAVKRSLGSL

Supplementary Figure S1. Deduced amino acid sequences of the recombinant proteins expressed in *E. coli.* The amino acid sequences are color coded, consistent with Figures 2A, 3A, 4A, and 5A. Blue, YKL-40; Pink, CHIT1; Light green, YKL-39.

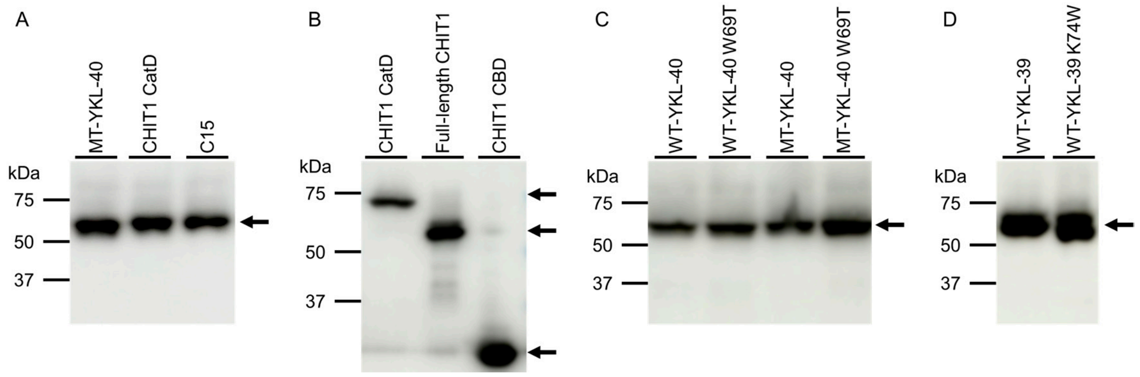

Supplementary Figure S2. Western blot analysis of the recombinant proteins using anti-V5 antibody. Arrow highlights the positions of the fusion proteins. (A) Expressed MT-YKL-40, CHIT1 CatD, and MT-YKL-40-CHIT1 CatD chimera (C15). (B) Expressed Full-length CHIT1, CHIT1 CatD, and CHIT1 CBD. (C) Expressed WT-YKL-40, MT-YKL-40, and their respective W69 mutants (W69T). (D) Expressed WT-YKL-39, and K74 mutations (K74W).

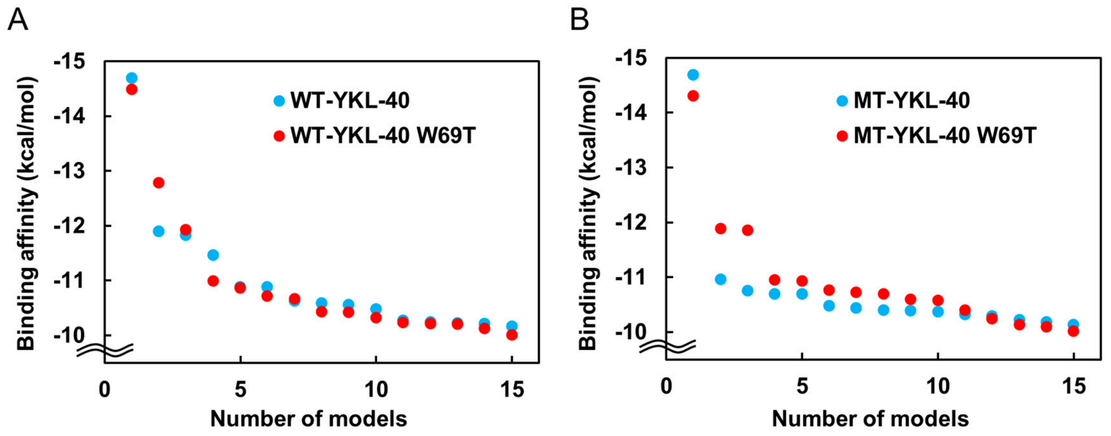

Supplementary Figure S3. Binding free energy distribution for WT-YKL-40, MT-YKL-40, and their respective W69T mutants. Lower binding free energy values indicate stronger and thermodynamically more stable binding interactions.(A) Binding free energy (kcal/mol) comparison between WT-YKL-40 (blue) and its W69T mutant (red) across the top 15 docking models. (B) Binding free energy (kcal/mol) comparison between MT-YKL-40 (blue) and its W69T mutant (red) across the top 15 docking models.

Supplementary Table S1. Combination of primer and template to prepare the recombinant proteins by PCR.

Product name	Template DNA	Primer name	Sequence (5' - 3')
CHIT1 CBD	Full-length CHIT1	CHIT1 CBD_Fw	GTACCCGGGGATCCTCCAGAG CTTGAAGTTCCAAAACCAG
		Sal_BGH_Rv	AGGGGTCGACTAGAAGGCACA GTCGAGGCTGATCA

WT-YKL-40 W69T product 1	WT-YKL-40	pEZZ18_Fw2533	CCGTGCTGTGTGCAGAACAGA GGG
		YKL-40_W69T_Rv	CATTCCACTCAGTGGTGTCGAT GTGATCGTTGCTTATATT
WT-YKL-40 W69T product 2	WT-YKL-40	YKL-40_W69T_Fw	CATCGACACCACTGAGTGGAA TGATGTGACGCTCTACGGC
		Sal_BGH_Rv	AGGGGTCGACTAGAAGGCACA GTCGAGGCTGATCA
↓			
WT-YKL-40 W69T	WT-YKL-40 W69T product 1+2	pEZZ18_Fw2533	CCGTGCTGTGTGCAGAACAGA GGG
		Sal_BGH_Rv	AGGGGTCGACTAGAAGGCACA GTCGAGGCTGATCA

WT-YKL-39 K74W product 1	WT-YKL-39	pEZZ18_Fw2533	CCGTGCTGTGTGCAGAACAGA GGG
		YKL-39_K74W_Rv	CACTCTTGTCCCAGATGATAAC CTTGTTGTTTTCGA
WT-YKL-39 K74W product 2	WT-YKL-39	YKL-39_K74W_Fw	GGTTATCATCTGGGACAAGAG TGAAGTGATGCTCTA
		Sal_BGH_Rv	AGGGGTCGACTAGAAGGCACA GTCGAGGCTGATCA
↓			
WT-YKL-39 K74W		pEZZ18_Fw2533	CCGTGCTGTGTGCAGAACAGA GGG

	WT-YKL-39 K74W product 1+2	Sal_BGH_Rv	AGGGGTCGACTAGAAGGCACA GTCGAGGCTGATCA
